# Comparison of the iStent Inject^®^ versus the iStent Inject^®^ W—Both in Combination with Cataract Surgery—In Open-Angle Glaucoma

**DOI:** 10.3390/jcm12237259

**Published:** 2023-11-23

**Authors:** Steffen Deneri, Ralph-Laurent Merté, Nicole Eter, Viktoria C. Brücher

**Affiliations:** Department of Ophthalmology, University Münster, 48149 Münster, Germany; ralph-laurent.merte@ukmuenster.de (R.-L.M.); nicole.eter@ukmuenster.de (N.E.);

**Keywords:** glaucoma, iStent inject, iStent inject W, intraocular pressure

## Abstract

We compare the short- and mid-term postoperative outcomes of the iStent inject^®^ with its successor, the iStent inject^®^ W. A retrospective monocentric study was performed to compare the iStent inject^®^ used for cataract surgery with the iStent inject^®^ W, also used for cataract surgery. The primary study endpoint was intraocular pressure (IOP) reduction six months after surgery. Six-month follow-up results were available for 35 eyes from 27 patients in the iStent inject^®^ group and for 32 eyes from 25 patients in the iStent inject^®^ W group. IOP reduction at six months post surgery was significantly greater in the iStent inject^®^ W group (−2.2 mmHg [iStent inject^®^ W] vs. −0.06 mmHg [iStent inject^®^], *p* = 0.037). There was a statistically greater decrease in glaucoma medication administration at six months in the iStent inject^®^ group than in the iStent inject^®^ W group (−1.28 agents vs. −0.62 agents, *p* = 0.007). These findings support the hypothesis that the superior positioning of the iStent inject^®^ W (due to its larger base diameter) compared to the iStent Inject^®^ leads to greater IOP reduction. Because of the short follow-up period, small study cohort, and differences in the number of glaucoma patients, the study results must be interpreted carefully.

## 1. Introduction

Minimally invasive glaucoma surgery (MIGS) has become a major focus of glaucoma surgery within the last few years. MIGS seems to accomplish the general aim of achieving a significant intraocular pressure (IOP) reduction while lowering operative risk [1]. Furthermore, MIGS is also characterized by a lower risk of postoperative complications, such as severe hypotony, compared with more invasive procedures such as the standard procedure trabeculectomy. In addition, MIGS is easier and more quickly learned than traditional glaucoma surgery [2,3].

The iStent^®^ (Glaukos, Laguna Hills, CA, USA) is an MIGS device that drains aqueous humor from the anterior chamber directly into the Schlemm’s canal, bypassing the trabecular meshwork [4]. It has a proven IOP-lowering effect [5,6] and a low operative risk [6,7]. The iStent^®^ is usually implanted after successful cataract surgery. In a 2015 study, the iStent^®^ combined with phacoemulsification proved to be superior to phacoemulsification alone in reducing IOP [8]. Two iStent injects^®^ are usually preloaded onto an inserter and can be injected into the Schlemm’s canal two to three clock hours apart [9,10]. Glaukos developed the third-generation iStent Inject^®^ W to achieve a finer positioning of the iStent^®^ during surgery. The second-generation iStent inject^®^ has an overall height of 360 µm with a base diameter of 230 µm, while the third-generation iStent inject^®^ W has the same height and a base diameter of 360 µm [9]. The iStent inject^®^ is used in patients with uncontrolled open-angle glaucoma and pseudoexfoliation glaucoma [11,12].

Studies comparing the second-generation iStent inject^®^ with the first-generation iStent^®^ report a significant IOP reduction overall and greater IOP reduction for the second-generation iStent inject^®^ than for the first generation iStent^®^ [13,14,15]. To date, there is no study comparing the iStent inject^®^ with the iStent inject^®^ W. This study compares the effect and safety of the iStent inject^®^ with that of the iStent inject^®^ W at the end of a six-month follow-up.

## 2. Materials and Methods 

### 2.1. Study Design

This was a single-center retrospective study comparing the effectiveness and safety of the iStent inject^®^ W to that of its predecessor, the iStent inject^®^, in patients with primary open-angle glaucoma (POAG), pigmentary glaucoma, and pseudoexfoliation glaucoma. A uniform study design, with identical inclusion and exclusion criteria for both groups and standardized study endpoints, was adopted. All surgeries were performed by three experienced glaucoma surgeons who are proficient in both the second and third generations of the iStent^®^. 

### 2.2. Patients and Assessments

All patients who underwent an iStent inject^®^ and cataract surgery, and those who underwent an iStent inject^®^ W and cataract surgery, performed between January 2019 and December 2020 at the University Eye Clinic, Muenster, Germany, were recruited for this study. Up until the end of November 2019, the iStent inject^®^ was used in combined cataract surgeries, and from December 2019, the iStent inject^®^ W was used in place of the iStent inject^®^ in all surgeries. Patients were excluded from this study if they had been on follow-up for less than six months. All patients included in this study had open-angle glaucoma, pigmentary glaucoma, or pseudoexfoliation glaucoma.

The following baseline data were collected from the patient clinical records of the participants: age, sex, previous glaucoma surgery, intraocular pressure, number of glaucoma medications, 30° perimetry, optical coherence tomography (OCT) of the Bruch membrane opening (BMO), and OCT of the retinal nerve fiber layer (RNFL). 

### 2.3. Cataract Surgery and iStent^®^ Implantation

All patients underwent regular phacoemulsification cataract surgery under general or topical anesthesia. Afterwards, an iStent Inject^®^ W or iStent Inject^®^ implantation was performed, with two stents inserted at 30° to 60° from each other [9,10].

### 2.4. Outcome Measurements

The primary study endpoint was a reduction in IOP at the end of a six-month follow-up. The secondary parameters were IOP reduction on day 1 post operation, difference in 30° perimetry after six months, difference in BMO-OCT and RNFL-OCT after six months, reduction in glaucoma medication administration at six months post operation, and postoperative complications.

### 2.5. Peri- and Postoperative Complications

Peri- and postoperative complications were assessed. Complications were defined as follows: infection, device explantation, device-related interventions, and hypotony of <5 mm Hg.

### 2.6. Statistical Analysis

Data were collected from Fidus electronic patient records (Arztservice Wente GmbH, Darmstadt, Germany), and statistical analysis was performed using the Statistical Package for the Social Sciences (SPSS), version 28 (IBM Inc., Armonk, NY, USA). Normal distribution of the data was tested using the Kolmogorov–Smirnov test. If the distribution was normal, the independent-samples *t* test was used to compare continuous variables. The Mann–Whitney U test was used for data with nonnormal distributions. For binomial variables, the chi-square test was used for larger numbers. Fisher’s exact test was used when the number in a cell was less than five. A binomial test was used for the between-group comparisons of the patients in the two study groups. For all tests, a *p*-value of 0.05 or less was considered statistically significant. Microsoft Word was used as the graphics program for creating electronic figures.

## 3. Results

From January 2019 to December 2020, 86 iStent inject^®^ implantations for cataract surgery and 89 iStent inject^®^ W implantations for cataract surgery were performed. Six-month follow-up data were available for 35 eyes from 27 iStent inject^®^ patients and 32 eyes from 25 iStent inject^®^ W patients; these were used for the statistical analysis. The main baseline clinical and demographic characteristics of the study participants are presented in Table 1. 

**Figure 1:**
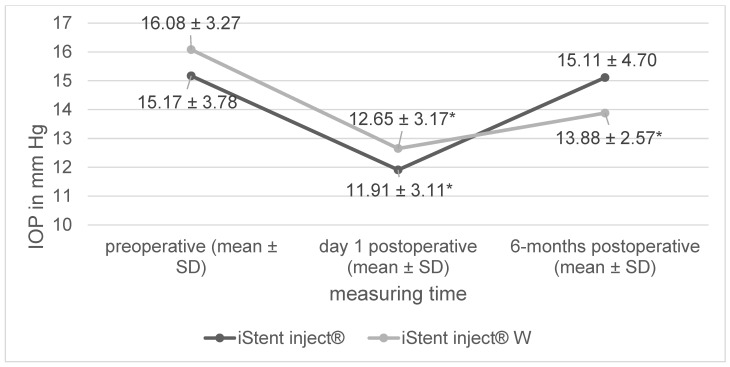
Change in intraocular pressure (* significant change compared to preoperative data, IOP = intraocular pressure, SD = standard deviation).

In both study groups, IOP immediately significantly decreased postoperatively on day 1, but increased after six months (Figure 1). The iStent inject^®^ W group showed a statistically significant reduction in IOP both immediately post operation (*p* < 0.01) and after six months (*p* < 0.01) when compared to the baseline IOP. In the iStent inject^®^ group, a significant reduction in IOP was observed only immediately after the surgery (*p* < 0.01), with no IOP reduction observed after six months (*p* = 0.94). In the between-group comparison, no significant difference in IOP reduction was observed postoperatively on day 1 (−3.43 mm Hg [iStent inject^®^ W] vs. −3.26 mm Hg [iStent inject^®^], *p* = 0.85). However, with respect to the primary study endpoint, there was a significantly greater reduction in IOP at the six-month mark in the iStent inject^®^ W group than in the iStent inject^®^ group (−2.2 mm Hg [iStent inject^®^ W] vs. −0.06 mm Hg [iStent inject^®^], *p* = 0.04).

**Figure 2:**
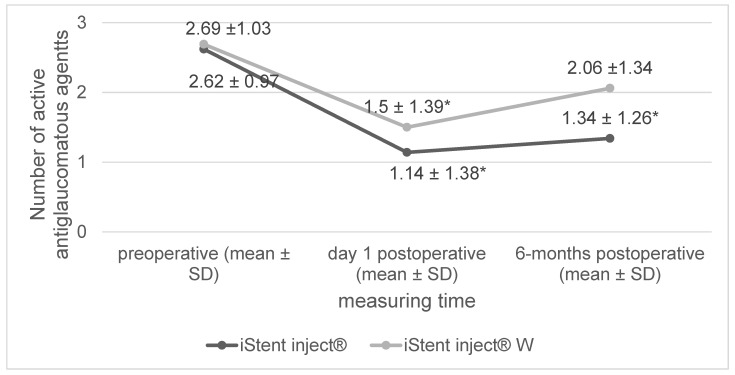
Changes in the number of antiglaucomatous agents (* significant change compared to preoperative data, SD = standard deviation).

The number of antiglaucomatous agents was significantly reduced post operation (Figure 2). At day 1 post operation, there was no significant difference between the iStent inject^®^ W group and the iStent inject^®^ group (−1.19 agents [iStent inject^®^ W] vs. −1.48 agents [iStent inject^®^] *p* = 0.26). After six months, the number of antiglaucomatous agents had increased in both groups. Overall, at the six-month mark, there was a greater reduction in the number of antiglaucomatous agents in the eyes in the iStent inject^®^ group than in those in the iStent inject^®^ W group (−0.63 agents [iStent inject^®^ W] vs. −1.28 agents [iStent inject^®^], *p* < 0.01). 

There were no significant differences in postoperative complications between the two study groups. Overall, complications were rare and not severe (Table 2). There was a single instance of vitreous prolapse due to cataract surgery in the iStent inject^®^ group. Four anterior chamber operations were necessary because of postoperative complications (two instances of anterior chamber hemorrhage, one iStent^®^ dislocation, one vitreous prolapse). In addition, one fortecortin injection was administered to treat Irvine–Gass syndrome. No further glaucoma surgeries were performed during the postoperative control interval.

Regarding the analysis of perimetry data, 23 eyes in the iStent inject^®^ group and 29 eyes in the iStent inject^®^ group were included in the analysis. Over the course of the six-month follow-up period, there was no significant difference in the changes in perimetry between the iStent inject^®^ W and iStent inject^®^ groups (main defects: +0.22 dB MD [iStent inject^®^ W] vs. +0.19 dB MD [iStent inject^®^], *p* = 0.67) (Table 3).

Regarding the analysis of BMO-OCT and RNFL-OCT data, 24 eyes in the iStent inject^®^ group and 29 eyes in the iStent inject^®^ group were included in the analysis. A comparison of the changes detected via BMO-OCT (−6 µm [iStent^®^ inject] W vs. +8 µm [iStent inject^®^], *p* = 0.24) and RNFL-OCT (−9 µm [iStent inject^®^ W] vs. −9 µm total [iStent inject^®^], *p* = 0.21) six months post operation also showed that there was no significant difference between the two groups. 

## 4. Discussion

This study compares the effect and safety of two minimally invasive glaucoma implants (the iStent inject^®^ and its successor, the iStent inject^®^ W) in patients with POAG, pigmentary glaucoma, and pseudoexfoliation glaucoma who underwent combined cataract surgery.

Both procedures (i.e., combined cataract surgery with the iStent inject^®^ vs. combined cataract surgery with the iStent inject^®^ W) yielded a significant decrease in IOP at day 1 post surgery, with a rebound in IOP at the end of a six-month follow-up period. Regarding the primary study endpoint (i.e., a reduced IOP at six months post operation), the iStent inject^®^ W produced a significantly stronger effect than the iStent inject^®^. The number of pressure-lowering drugs administered to the patients in both study groups was reduced over the course of the six-month follow-up period. However, there was a stronger reduction in the number of pressure-lowering drugs administered in the iStent inject^®^ group than in the iStent inject^®^ W group. Regarding surgical side effects, there were instances of anterior chamber hemorrhage, Irvine–Gass syndrome, vitreous prolapse, and an iStent^®^ dislocation, with no between-group differences observed. There were no severe surgical complications in either study group.

No other glaucoma surgeries were performed during the six-month follow-up period. All subsequent glaucoma surgeries occurred after the follow-up period. 

There are no published studies on the iStent inject^®^ W as of the time of writing this paper. In a study by Craven et al., only mild postoperative complications, such as iStent^®^ displacement (2.6%) or iStent^®^ obstruction (4.3%), were observed [16]. In a Samuelson et al. study, iStent^®^ obstruction occurred in 4% of the operations [17]. In several other studies, anterior chamber hemorrhage occurred in 2.5–70% of the procedures—the latter figure (i.e., 70%) includes even very mild cases of anterior chamber hemorrhage [18,19]. A mild hyphema is often a sign of blood reflux from the iStent^®^, which indicates correct positioning and typically resolves after a week [4].

A systematic review with a meta-analysis that analyzed the effect of various iStent^®^ devices demonstrated a significant IOP reduction even without combined cataract surgery [20]. A comparison of the iStent inject^®^ with combined cataract surgery versus the Hydrus with combined cataract surgery showed no difference in IOP reduction [21]. Compared to transluminal trabeculotomy with combined cataract surgery, the iStent inject^®^ with combined cataract surgery yielded a lower IOP reduction [22].

A direct comparison of the second-generation iStent inject^®^ with the first generation iStent^®^ revealed that the newer iStent inject^®^ yielded a significantly greater IOP reduction. The authors of the comparison study attribute this difference to the updated design of the newer device and the fact that two iStent injects^®^ were implanted compared to only one iStent^®^ [13]. Although there are several studies comparing the first and second generations of the iStent^®^, there has been no study comparing the second-generation iStent inject^®^ with the third-generation iStent inject^®^ W. 

Both the second- and third-generation iStents^®^ have the same mode of action and are made of the same material; the opening sizes are also identical. The second- and third-generation iStents^®^ differ only in the size of the base. The larger base in the iStent inject^®^ W is intended to ensure improved positioning [9]. However, the larger base of the iStent inject^®^ W may also cause lumen occlusion and slower overgrowth than the second-generation iStent inject^®^. Our data show a lower IOP in the iStent inject^®^ W group for a longer period than in the iStent inject^®^ group, supporting this hypothesis. 

Postoperatively, after both surgeries, some patients showed an increase in parameters measured via BMO-OCT or RNFL-OCT, which was significant in some cases. Such a change after glaucoma surgery is also known as reversed cupping and is related to a postoperative reduction in IOP [23,24,25]. 

This study has several limitations, primarily due to its retrospective nature. Randomized patient recruitment was not performed. At the University Eye Clinic, Muenster, Germany, the iStent inject^®^ was used in combined cataract surgeries until the end of November 2019 and was displaced by the iStent inject^®^ W as the device of choice starting in December 2019. Therefore, the two study groups were not randomized controlled, and the baseline data were not sufficiently comparable. Most notably, there were differences between the participants in the two groups regarding previous operations and perimetry data.

Furthermore, the study data (surgical method, IOP, perimetry, BMO-OCT, RNFL-OCT, and complications) were retrospectively extracted from the patients’ medical records. A further limitation is the lack of time points for IOP measurements. In particular, long-term data (more than a year) could not be included because there were insufficient follow-up data available. Several patients could not be included in the analysis because six-month follow-up data were not available. This is because of the retrospective design of the study and the fact that many patients have their postoperative follow-up performed by an outpatient ophthalmologist and are therefore not available for evaluation at the clinic. This limits the number of eyes evaluated. 

Patients with POAG, pigmentary glaucoma, and pseudoexfoliation glaucoma were recruited for this study. A conclusion for patients with POAG only is therefore limited. In addition, local glaucoma therapy was not standardized or controlled in either group. Glaucoma medication was administered individually to each patient based on clinical considerations.

Another limitation of this study is that a greater decrease in administered glaucoma medication was observed in the iStent inject^®^ group than in the iStent inject^®^ W group. Therefore, this could partially explain the weaker effect of the iStent inject^®^ with respect to IOP reduction, and would not indicate an inferiority of the device vis-à-vis the iStent inject^®^ W.

Overall, MIGS, such as the iStent inject^®^ and the iStent inject^®^ W, achieves a mild or moderate IOP reduction with low perioperative risk, and is primarily recommended for patients undergoing cataract surgery who have open-angle glaucoma that is not significantly advanced. When a significantly large IOP reduction is needed in patients with very high IOP or advanced glaucoma, standard glaucoma surgery (e.g., trabeculectomy) is typically recommended [5,6,7].

This study focused on iStent^®^ surgery in combination with cataract surgery. Therefore, a comparison of iStent inject^®^ vs. iStent inject^®^ W as standalone procedures was not performed. There was also no comparison of the iStent inject^®^ with cataract surgery versus cataract surgery alone. This has already been investigated in a meta-analysis conducted in 2015 [8].

In summary, both the iStent inject^®^ and the iStent inject^®^ W, as minimally invasive procedures, have a low surgical risk. These procedures combine well with cataract surgery. In our study, the iStent inject^®^ W yielded a greater IOP reduction after six months. Broadly, it must be mentioned that there was a relatively short follow-up period of only six months and a small study cohort. Therefore, any conclusions about the superiority of the iStent inject^®^ W over its predecessor, the iStent inject^®^, should be made very carefully. Findings based on longer-term data are needed.

## Figures and Tables

**Table 1:** Baseline characteristics.

	iStent Inject^®^ (Mean ± SD)	iStent Inject^®^ W (Mean ± SD)	*p*-Value
*n*	35	32	0.40
age	69.86 ± 6.85	70.20 ± 10.30	0.87
sex			
M	17 (48.5%)	13 (40.6%)	0.62
F	18 (51.5%)	19 (59.4%)	0.63
number of previous operations	selective laser trabeculoplasty: 2 (5.7%)	selective laser trabeculoplasty: 13 (40%)	<0.01 *
	cyclophotocoagulation: 1 (2.9%)	cyclophotocoagulation: 0	0.93
	trabeculectomy: 1 (2.9%)	trabeculectomy: 0	0.93
visual field (MD)	11.76 ± 2.3 dB	6.15 ± 5.32 dB	0.04 *
RNFL-OCT	175.9 ± 53.1 µm	191.6 ± 39.6 µm	0.25
BMO-OCT	187.6 ± 72.1 µm	211.6 ± 79.9 µm	0.41
preoperative IOP	15.17 ± 3.78 mm Hg	16.08 ± 3.27 mm Hg	0.29
number of glaucoma agents	2.63 ± 0.97	2.69 ± 1.03	0.73

*n*: number of eyes, M: male, F: female, MD: main defects, SD: standard deviation, * significant result, dB: decibel, IOP: intraocular pressure.

**Table 2:** Postoperative complications.

Postoperative Complications	iStent Inject^®^	iStent Inject^®^ W	*p*-Value
anterior chamber hemorrhage	0	2 (6.2%)	0.13
dislocation of iStent^®^	0	1 (3.1%)	0.29
postoperative hypotony (<5 mm Hg)	0	0	>0.99
Irvine–Gass syndrome	0	1 (3.1%)	0.48

**Table 3:** Changes in perimetry: a between-group comparison of BMO-OCT and RNFL-OCT data (MD = main defects).

	iStent Inject^®^ (Mean ± SD)	iStent Inject^®^ W (Mean ± SD)	*p*-Value (Difference between Groups)
Perimetry: preoperative (MD)	−11.75 ± 11.02 dB	−6.15 ± 5.32 dB	
Perimetry: 6 months post operation (MD)	−11.56 ± 11.58 dB	−6.37 ± 6.34 dB	0.67
BMO: preoperative	188 ± 71 µm	212 ± 80 µm	
BMO: 6 months post operation	183 ± 67 µm	218 ± 92 µm	0.24
RNFL: preoperative (global)	176 ± 53 µm	192 ± 40 µm	
RNFL: 6 months post operation (global)	184 ± 53 µm	200 ± 37 µm	0.21

## Data Availability

The data presented in this study are available on request from the corresponding author. The data are not publicly available due to patient privacy.
